# 

**Published:** 1866-11

**Authors:** 


### ORIGINAL CONTRIBUTIONS.

ART. XXXVIII. Report on Cerebro-Spinal Meningitis. By J. S. Jewell, M.D., Prof. Anatomy, Chicago Medical College; Member of Illinois State Medical Society, etc. (Read to the Illinois State Medical Society, June, 1866,) ... 658

ART. XXXIX. Typho-Malarial Fever. By Wm. H. Veatch, M.D., Pawnee, Sangamon Co., Ill. (Read to the Ill. State Medical Society, 666

ART. XL. Epidemic Diseases. By F. B. Haller, M.D., Vandalia, Ill. (Read to the Illinois State Medical Society.) ... 679

ART. XLI. Early Diagnosis of the Backward Curvature of the Spine. By Julien S. Sherman, M.D., Chicago. ... 683

### BOOK NOTICES.

A Practical Treatice on the Physical Exploration of the Chest, and the Diagnosis of the Diseases of the Respiratory Organs. By Austin Flint, M.D., Prof. Prin. and Prac. of Medicine in the Bellevue Hospital Medical College, etc., etc. Second edition revised, ... 686

Elements of Medical Chemistry. By R. Howard Rand, M.D., Prof. of Chemistry in the Jefferson Medical College, ... 686

A Manual of Blow-Pipe Analysis and Demonstrative Mineralogy By Wm. Elderherst, M.D., Prof. of Chemistry in the Rensselaer Polytechnic Institute. Third edition, revised and greatly enlarged, ... 686

### EDITORIAL.

Death of Professor Daniel Brainard, ... 687

Meeting of the Medical Profession.—Death of Daniel Brainard, M.D., ... 688

Eulogy by Dr. Brock McVickar, ... 689

Meeting of the Sangamon County Med. Society.—Resolutions of Respect, 691

Mortality in Chicago for the Month of September, ... 692

Easily Frightened, ... 692

